# A Community Resource Map to Support Clinical–Community Linkages in a Randomized Controlled Trial of Childhood Obesity, Eastern Massachusetts, 2014–2016

**DOI:** 10.5888/pcd14.160577

**Published:** 2017-07-06

**Authors:** Lauren Fiechtner, Gabriella C. Puente, Mona Sharifi, Jason P. Block, Sarah Price, Richard Marshall, Jeff Blossom, Monica W. Gerber, Elsie M. Taveras

**Affiliations:** 1 Division of Gastroenterology and Nutrition, Massachusetts General Hospital for Children, Boston, Massachusetts; 2 Division of General Academic Pediatrics, Department of Pediatrics, Massachusetts General Hospital for Children, Boston, Massachusetts; 3 Columbia University, the College of Physicians and Surgeons, New York, New York; 4 Section of General Pediatrics, Yale University School of Medicine, New Haven, Connecticut; 5 Obesity Prevention Program, Department of Population Medicine, Harvard Medical School and Harvard Pilgrim Health Care Institute, Boston, Massachusetts; 6 Harvard Vanguard Medical Associates, Boston, Massachusetts; 7 Center for Geographic Analysis, Harvard University, Cambridge, Massachusetts; 8 Department of Nutrition, Harvard School of Public Health, Boston, Massachusetts

## Abstract

**Background:**

Novel approaches to health care delivery that leverage community resources could improve outcomes for children at high risk for obesity.

**Community Context:**

We describe the process by which we created an online interactive community resources map for use in the Connect for Health randomized controlled trial. The trial was conducted in the 6 pediatric practices that cared for the highest percentage of children with overweight or obesity within a large multi-specialty group practice in eastern Massachusetts.

**Methods:**

By using semistructured interviews with parents and community partners and geographic information systems (GIS), we created and validated a community resource map for use in a randomized controlled trial for childhood obesity. We conducted semistructured interviews with 11 parents and received stakeholder feedback from 5 community partners, 2 pediatricians, and 3 obesity–built environment experts to identify community resources that could support behavior change. We used GIS databases to identify the location of resources. After the resources were validated, we created an online, interactive searchable map. We evaluated parent resource empowerment at baseline and follow-up, examined if the participant families went to new locations for physical activity and food shopping, and evaluated how satisfied the families were with the information they received.

**Outcome:**

Parents, community partners, and experts identified several resources to be included in the map, including farmers markets, supermarkets, parks, and fitness centers. Parents expressed the need for affordable activities. Parent resource empowerment increased by 0.25 units (95% confidence interval, 0.21–0.30) over the 1-year intervention period; 76.2% of participants were physically active at new places, 57.1% of participant families shopped at new locations; and 71.8% reported they were very satisfied with the information they received.

**Interpretation:**

Parents and community partners identified several community resources that could help support behavior change. Parent resource empowerment and use of community resources increased over the intervention period, suggesting that community resource mapping should inform future interventions.

## Background

Novel approaches to care delivery that leverage clinical and community resources and address sociocontextual factors could improve outcomes for children at high risk for obesity. Increasing physical activity and improving nutrition (1) can improve children’s weight and well-being. However, clinical obesity interventions targeted at modifying these behaviors have had limited success (2–4). Lack of information about health-promoting resources in communities may have limited the success of these approaches.

Mapping tools such as geographic information systems (GIS) can be used to describe community resources, including access to fresh fruit and vegetables (5), public health services (6), and resources for diabetes and related conditions (7). Interventions for adults designed to promote physical activity have incorporated community resource guides (8,9). Equipping providers with these community resource guides led to enhanced provider counseling about exercise, referrals to community programs, and increased patient physical activity (9). Mapping tools could be used to create searchable online community resource maps for providers and families.

## Community Context

Few articles (10) have described the process of creating resource maps and few have engaged parents and stakeholders in the process of choosing the resources. No community resource map to our knowledge has targeted resources for childhood obesity and none have covered an area as large as eastern Massachusetts, which has a population of 4 million, an area of 6,000 km^2^, and a mix of rural and urban areas. The objective of this article is to describe the process by which we created an online interactive health resources map for use in the Connect for Health randomized controlled trial for treatment of childhood obesity, the process of validating the resources identified, and evaluation of the usefulness of the map by examining changes in parent resource empowerment (knowledge and ability to access resources) (16), use of community resources, and participants’ satisfaction with the information they received about resources in their community. We hypothesized that parental resource empowerment and use of community resources would increase with the use of community resource mapping and that participant families would be satisfied with the tailored information provided to them.

## Methods

We conducted resource mapping for use in the Connect for Health randomized trial (11). The overall goal of the trial was to develop novel approaches to care delivery that leverage community resources and address sociocontextual factors to improve family-centered childhood obesity outcomes. We first interviewed parents of children who were successful at getting to a healthier weight and community partners to identify the community resources for the map and then implemented the map among Connect for Health participants.

We had a 2-step process for creating maps. First, we conducted structured interviews of parents and sought out stakeholder and expert feedback to determine what types of resources should be included. Second, we initiated a validation process for fitness centers and supermarkets.

For the structured interviews with parents we purposively recruited parents whose children were already participating in child focus groups designed to help inform the intervention. These parents had children who were seen for well-child care at Harvard Vanguard Medical Associates, the multi-specialty, multi-site practice where the Connect for Health intervention took place, and were identified as positive outliers because they had succeeded in improving their body mass index (BMI) (as defined by having a negative BMI *z*-score slope for up to the 5 years prior) despite living in obesity hotspots (ie, zip codes with >15% prevalence of childhood obesity) (12).

For the purposes of this study, the sample was limited to parents of children who were aged 10 to 12 years at the time of study recruitment in February 2014 (n = 193) and had maintained a negative BMI *z*-score slope through October 2013 (n = 174). The institutional review boards of Partners Health Care approved the study protocol.

Two families opted out of participation; we ranked the remaining 172 children in our recruitment sample by BMI *z*-score slope. Parents of children with the most negative slopes were contacted for recruitment first. All 172 parents were called, 36 (21%) agreed to participate, 21 (12%) brought their children to the focus groups on the day of the interview, and we interviewed 11 (6%) of these parents, again prioritizing those parents of children with the most negative BMI *z*-score slope.

We conducted 45-minute semistructured interviews with 11 parents while their children attended focus groups. As part of the interviews we showed families a mock-up of the map. We asked parents for their input on what barriers and facilitators existed when trying to help their children achieve a healthier weight and what resources and functions they would find helpful in a community resource map. Two research staff members (S.P., L.F.) conducted the parent interviews, and we provided parents with $30 for their participation.

All parent interview sessions were audio-recorded and transcribed by an independent transcription company. After transcription, 2 members of the research team (L.F., G.P.) analyzed data using the immersion-crystallization method (13). This method entailed independently reading and analyzing transcripts. L.F. and G.P. then met to discuss their independent analyses and identify emerging themes and representative quotes. After a list of themes was developed and definitions were clarified, transcript texts were coded line by line. The list of themes was modified as new themes emerged and links between themes were made. Analysis was considered complete when no new themes were generated from transcript review and discussion. Ultimately, L.F. and G.P. reached consensus on the final list of themes and representative quotes.

To choose the most effective and useful resources, we also consulted with community partners with knowledge of resources: representatives from the YMCA of Greater Boston, Appalachian Mountain Club, the Metropolitan Area Planning Council, and *ChopChop* magazine. We also sought feedback from 2 pediatricians and 3 obesity researchers who focus on the built environment, or the man-made surroundings that provide the setting for human activity (for example, the places for physical activity and food consumption in a child’s neighborhood). We also showed community partners a mock-up of the map. We asked what resources and functions they would suggest we put on the map. These stakeholder feedback sessions were semistructured and lasted 30 minutes to 1 hour.

Parents, community partners, and experts identified several resources they felt would be useful, including farmers markets, social support resources, supermarkets, and fitness centers. We located farmers markets and social support resources on our own via internet search on the Commonwealth of Massachusetts website (mass.gov). We gathered information on locations of parks and other green spaces from Mass GIS (http://www.mass.gov/anf/research-and-tech/it-serv-and-support/application-serv/office-of-geographic-information-massgis/), a State of Massachusetts GIS database. In a separate process, we identified fitness centers and supermarkets from a large commercial database, Dun and Bradstreet (Esri Business Analyst, 2013), because it is the database used by ESRI Business Analyst 2013, which is a data set purchased every year by Harvard for use by all affiliates. We validated supermarkets and fitness centers provided by Dun and Bradstreet because prior studies have shown that large commercial databases can be prone to misclassification error (14,15). Open space and parks included in such commercial databases are likely to be valid because these variables are obtained from Mass GIS, compiled from existing town assessor maps and verified by aerial photography. From the 2013 Dun and Bradstreet database, we validated fitness centers and supermarkets located in the 5 zip codes that had the highest prevalence of obesity for each of the 6 intervention sites. Of the 2,971fitness centers identified in eastern Massachusetts, 182 were in these zip codes. Of the 2,264 supermarkets identified in eastern Massachusetts, 341 were in these zip codes. To validate fitness centers and supermarkets we called these businesses 3 times on separate days and at different times. If we did not reach them after the 3 attempts or if their telephone number was invalid, we did an internet search for the business name or address. If the information provided on the internet demonstrated the location was still open and that it was either a supermarket or a fitness center we included the location on the map. If we reached the business we confirmed whether or not it was a supermarket (defined as selling a variety of products including fresh fruit and vegetables) or a fitness center (defined as offering a form of physical activity for either children or adults). We also confirmed the name, address, and telephone number with the business and made changes as needed. Finally, we asked the valid fitness centers if they provided programs for children.

Before the map went live, we had our health coaches use the map in practice visits on a production server to ensure the map was easy to use and all functions were working. All difficulties were demonstrated and communicated to our colleagues at the Center for Geographic Analysis who created the web map. After α testing was complete the web map was placed on a public server.

The map was tested and evaluated during the Connect for Health trial. By using electronic health record data and GIS we identified the 6 pediatric practices that cared for the highest percentage children with overweight or obesity within the 14 pediatric practices at Harvard Vanguard Medical Associates in eastern Massachusetts. Inclusion criteria for the trial were that the child was aged 2 to 12.9 years, had a BMI at or above the 85th percentile, and received their routine health care at the 6 practices. Pediatricians referred 1,485 children who were eligible; 721 (49%) children were recruited. Of the 721, 664 (92%) children had BMI measurements in their medical records, and 657 of the 721 (91%) children completed the survey we conducted at the end of the study. Children were randomized to 1 of 2 arms: 1) enhanced primary care, which consisted of flagging children with BMI at or above the 85th percentile, clinical decision support tools for pediatric weight management, parent educational materials, a community resource guide, and monthly text messages (n = 361), or 2) enhanced primary care plus contextually tailored, individual health coaching (twice-weekly text messages and telephone or video contacts every other month) to support behavior change and linkage of families to neighborhood resources via the community resource map (n = 360). All participants had access to email. Recruitment began in July 2014 and the 1-year intervention and data collection was completed in June 2016.

Parental resource empowerment was assessed at baseline and follow-up via parents’ completion of the child weight management subscale of the parent resource empowerment scale (16). Previous research shows that this scale demonstrates high internal consistency (internal reliability score of α = 0.96) (16,17) and is sensitive to change (17). The 5 items in the scale assess parents’ perceived knowledge of resources, ability to access resources, comfort with accessing resources, knowledge of how to find resources, and ability to acquire resources. Response options are 1, strongly disagree; 2, disagree; 3, agree; or 4, strongly agree. Items were averaged to create a summary parental empowerment score. Cronbach’s α for this score was 0.87 (18). We used generalized linear repeated measure models to account for clustering within each participant over time. Although 657 (90%) of participants completed the follow-up survey, we used intention-to-treat analysis and multiple imputation for all 721 participants.

Use of physical activity resources and food establishments was assessed at baseline and follow-up via survey. At baseline parents were asked the top 3 places where their child was physically active and the top 3 places they purchased food for their family. At follow-up we asked if the child was active at new places and if they went to new establishments to purchase food for their family over the past year; 638 families answered these questions. We used χ^2^ tests to assess the difference between the intervention arms.

Of the 657 families that completed the follow-up survey 496 (75%) of participating families confirmed receiving information on community resources. To examine satisfaction with the resources provided, these 496 families were asked at follow-up, “how satisfied were you with the information you received about resources in your community?” All statistical analysis was completed in SAS 9.4 (SAS Institute). We used χ^2^ tests to assess the difference between the intervention arms.

## Outcome

Of the 11 participants in the parent interviews, 9 were the child’s mother and 2 were the child’s father. Nearly two-thirds spoke primarily English at home (100% were fluent in English), 82% had completed at least some college, 45% self-identified as Hispanic or Latino, and 45% self-identified as black. Participants in the Connect for Health trial were also racially and ethnically diverse with 33% identifying as black and 22% identifying as Latino. At baseline, 51% of parents of Connect for Health participants were college graduates.

Interviewees and community partners indicated they wanted a community resource map that included physical activity resources such as parks and playgrounds, fitness centers, walking trails, pools, ice skating rinks, YMCAs, Boys and Girls Clubs of America (Table 1). They thought that nutrition resources such as supermarkets and farmers markets would be useful to include. Social support programs such as the Supplemental Nutrition Program for Women, Infants, and Children locations, Department of Transitional Assistance offices, and food pantries were also mentioned as important resources. Modes of transportation such as subway and bus stops were also mentioned. Finally, interviewees and community partners reported that street views of the map’s resources would be helpful to allow families to review the condition and safety of the locations. They also wanted map functionality that would provide directions and enable a printable resource guide.

**Table 1:** Resources Identified by Parents of Positive Outliers^a^, Community Partners as Beneficial for Weight Management, Connect for Health Randomized Controlled Trial of Childhood Obesity, Eastern Massachusetts, 2014–2016

Resource	Type
Physical activity	•Bike trails •Parks •Playgrounds •Fitness centers •Schools •Walking trails •Pools •Recreational centers •Open space •Canoe launch points •Ice skating rinks •YMCAs •Community gardens •Boys and Girls Clubs
Nutrition	•Supermarkets •Farmers markets •Farms
Social support services	•Supplemental Nutrition Program for Women, Infants, and Children (WIC) offices •Department of Transitional Assistance offices •Food pantries

a Children who succeeded in improving their body mass index.

The parents of positive outlier children we interviewed stressed the benefit of affordable, high quality, and nearby opportunities for physical activity and nutritional food in helping their child achieve a healthier weight (Table 2). One mother described the need for affordable options this way: “I think more things that are free, because me being a single mother . . . I’m always looking for something. If it costs too much you can’t go.” They also said that support from families, neighborhoods, communities, and schools was invaluable. One mother noted that community support was important: “I think the community itself — they encourage kids to do sports.”

**Table 2:** Interview Domains for Parents of Positive Outliers^a^ and Representative Quotes, Connect for Health Randomized Controlled Trial of Childhood Obesity, Eastern Massachusetts, 2014–2016

Domain	Quote
**Family-level factors**	**Budget constraints for physical activity resources**
	•I think more things that are free, because me being a single mother is like I’m always looking for something. If it costs too much you can’t go. •I think — activities that are free — or reduced cost, you know, affordable I should say. Not necessarily free, but affordable.
	**Budget constraints for healthy food**
	•Because if you think about it, when you go to the grocery store, they have Little Debbie cakes for $1.19. If you want to buy something healthy, it’s almost $5.00. •It’s healthy food, but it’s expensive.
	**Health education is important**
	•The first thing you have to do is educate the parents to get the healthy food for their family. •To tell you the truth, I really do not know if it will be helpful (referring to the community resource map), because to me, is to educate first. To inform — that helps.
	**Time constraints**
	•That’s where he’s been struggling because we haven’t been able go to gyms because of the school, the time we go to church. We go to church three times a week, so when he comes from school, we do his homework and then around 6:30 pm, 7:00 pm, we go to church. The dates that we don’t have church, we just stay home. •It’s been difficult to get them to go, because by the time they get home from school, and they did their homework and stuff, and they’re ready to go, everybody’s exhausted. “Oh, mommy, it’s time to cook dinner, and then let’s go to bed.”
**School environment (can be both beneficial and harmful)**	•Of course, at school, the meal is balanced at school, so we do it at home, but at school also. It was a big help all together. •When they are in the house, you know what they’re doing, but once they go out — she eats in the school at times. We say to her, “You know, you can eat, but watch what you’re eating.” At times the kids will have some ice creams, candies. That’s where, you know? At times it becomes really tough to control them. •The cafeteria at school, I don’t think they really give the best option for food for kids. I think the best thing to do sometimes, is to bring your own lunch, instead of having what they offer at school.
**Neighborhood-level factors**	**Access to healthy food**
	•Even where we live, they have a farmers market on Thursdays, and everybody sees it there because it is right *there*. •It’s hard sometimes, but I’m always looking for the specials and going to different places, because you see the different prices. Even if you have to waste a little bit of gas, you might as well go the extra mile and say, “Let me buy the oranges and fruits and vegetables right here, because it’s much cheaper.” You waste your gasoline, but — it’s better, at the end, because you save some money.
	**Access to unhealthy food**
	•At the beginning it was not easy ‘cause we have this McDonald’s close, and then the other [children] wanted to go eat there. •You have to be careful because we have a lot of supermarkets around. It’s up to you what you want to buy. I know people, they just buy the greasy food. •Fast food, ‘cause it’s everywhere. It’s like, “Oh, I’m hungry. I just left school. I wanna eat.”
	**Access to outdoor space**
	•When the spring comes up, yeah, they'll go to the park, play soccer, or they'll do Frisbee — her and her sister. . . . We have a park right across the street. •The back yard, and just take an hour, an hour and a half jumping rope. It doesn’t matter what it is, really. It’s just keeping the body active. He doesn’t have to go to a specific place.
	**Neighborhood and community support**
	•I think the community itself — I mean, encourage, of course — they encourage kids to do sports. •Also we go to church. He’s involved in basketball, too, and they have activities outside. •Having a good neighborhood also makes you healthier.
	**Safety concerns**
	•We don’t allow him to go outside by himself because so many things happening around and you don’t know. Sometimes we don’t know the neighbor and it’s hard because we should know who’s there and who’s not there. •With my kids, I’m always concerned about who’s living next door or who’s around the neighborhood. That way you know where to go and not to. Because you know so many things are happening and things happen in a flash.
**Benefits of a community resources map**	**Finding resource can be time consuming**
	I know there are resources, but it’s hard to find them. Maybe if there were easier resources for parents to get to know, that didn’t require them to have to spend all the time. I think it would be a lot different for a lot of families. I really do.
	**Map provides the opportunity to learn about new resources and plan activities**
	•Yeah, because I didn’t even know that there was a free gym on Saturdays right down the street, where kids play. I didn’t even know it was there. •Yeah, I think having this will really help you to plan, “Okay, I’m done with this. Oh, where next?” •Sometimes we don’t know that it’s there and it’s very close and we could go even walking or driving — riding a bike. It’s very helpful.
	**Ease of online format**
	•When I found things in my community . . . I went online and signed up for something. •It’s more effective and it’s more handy because these days everything is online, everyone is using smart phones.
	**Search capabilities of map**
	•Search for it. There’s a lot of farmers markets around and even in the regular stores, like I mentioned. Search for it. I mean search and go for it and go and buy vegetables and go buy the things we need at home to get a healthy life. •Look, ‘cause the resources are out there, if they just look.

a Children who succeeded in improving their body mass index.

Parents identified poor access to healthy food and easy access to fast food as barriers to a healthy weight in their community. Parents noted time constraints and safety issues as barriers to accessing community resources as well as a general lack of information. Parents said a community resource map or a list of resources would make it easier for them to identify locations to help them with their children’s health.

Parents indicated a community resource map would permit them to learn about new options in their community. They said many parents did not know about the healthy resources in their communities, and having 1 centralized database would be useful. They also felt that having the knowledge conveyed through the health coach would be helpful and could bring children together. Finally, they appreciated that they could search on the map for specific resources such as farmers markets.

Among the 341 supermarkets in the Dun and Bradstreet database that we called, 94 (28%) were actually supermarkets. Many (24%) were convenience stores. Others were sandwich shops, meat markets, liquor stores, pizza shops, restaurants, or ethnic-food stores that did not sell fruits or vegetables, and bakeries. Seventy-seven (42%) of the 182 fitness centers we called were actually fitness centers. Forty-five (63%) of the 71 valid fitness centers that we could reach by telephone offered programs for children. Many of the invalid establishments were nail spas, private homes, or no longer open.

The validated resources were placed on an interactive online web platform that is also mobile friendly. Types of resources can be clicked on or off so that health care professionals or families can view all types of resources in their neighborhood or just the type they are looking for. The map is 1 large map (Figure), and users can zoom into their address or neighborhood. The map also offers walking directions to the locations, a search function, and a printable map with the list of the locations, telephone number, address, and other attributes such as dates and times the resource is open.

**Figure:**
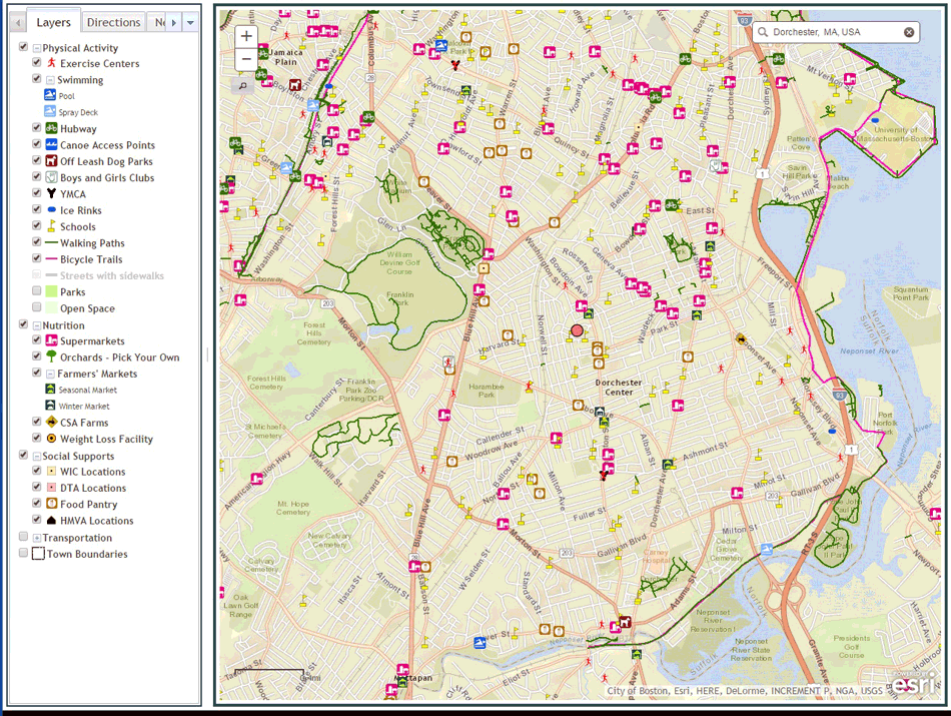
The online community resource map developed for the Connect for Health randomized controlled trial of childhood obesity.

Overall parent resource empowerment increased by 0.25 points (95% confidence interval [CI], 0.21–0.30) over the 1-year intervention period. We found no significant difference between those who received the online interactive map during health coach counseling and those who received the community resource guide (Table 3). Over the 1-year intervention period, 74.6% of those who received the interactive map and 77.7% of those who received the community resource guide went to new physical activity locations (*P* = .36); 57.1% of participants went to a new place to purchase food (55.9% of those who received the map and 58.2% of those who received the resource guide [*P* = .55]). Overall, 71.8% of participants (75.8% of those who received the map and 65.6% of those who received the resource guide [*P* = .01]) said they were very satisfied with the resources provided.

**Table 3:** Changes in Parent Resource Empowerment^a^ From Initial Visit to 1-Year Follow-up by Study Arm and Combined (N = 721)^b^, Connect for Health Randomized Controlled Trial of Childhood Obesity, Eastern Massachusetts, 2014–2016

Study Arm	Mean (Standard Deviation)		Mean Change (95% Confidence Interval)	β Value (95% Confidence Interval), Difference
	Baseline	1-yr Follow-up		
Enhanced primary care plus health coaching (received map)	3.0 (0.5)	3.2 (0.6)	0.22 (0.15 to 0.28)	0.07 (−0.02 to 0.16)
Enhanced primary care (received community resource guide)	2.9 (0.5)	3.1 (0.6)	0.29 (0.22 to 0.35)	1 [Reference]
Combined	2.9 (0.6)	3.2 (0.6)	0.25 (0.21 to 0.30)	Not applicable

a Knowledge and ability to access resources assessed by child weight management subscale of the parent resource empowerment scale (16).

b Intention-to-treat analysis (using multiple imputation).

## Interpretation

We found that resources for nutrition, physical activity, and social supports were important to parents of children who had succeeded in improving their BMI despite living in obesity hotspots. Community partners and built environment–obesity experts also believed that these resources were important to provide. We also found that families wanted affordable and convenient options for nutrition and physical activity. As has been demonstrated in other built environment research, families found that supermarkets were facilitators in getting to a healthier weight and easy access to fast-food restaurants was a barrier (19,20). They also found parks and playgrounds to be helpful, as demonstrated in previous studies (21,22). After determining what types of locations were important, we gathered resources to create the maps. We found validation of supermarkets and fitness centers to be necessary. Many of the supermarkets and fitness centers provided by Dun and Bradstreet 2013 proved invalid, as in previous studies (14,15). Parent resource empowerment and use of resources increased over the intervention period among study participants, although they did not differ by whether participants received the online interactive map or the mailed community resource guide. Most families were very satisfied with the information on community resources they received, and those who received the map were significantly more satisfied than those who received the community resource guide.

We were surprised that those with access to the interactive map did not increase their resource empowerment more or use more resources than those who received the resource guide. This finding suggests that providing a list of resources in a participant’s or patient’s community is equally as effective as an online, interactive, searchable map. Although families who received the map were more satisfied with the resources they received, the ease of use of a paper copy may be just as beneficial as the more sophisticated GIS map.

Other studies have used GIS to map community resources. The University of Chicago and Kaiser Permanente used GIS to aid with community health needs assessments, including examining access to parks and fast-food restaurants (23,24). However, to our knowledge, no study before ours has designed a map for a randomized controlled trial of childhood obesity, and no other program has mapped such a large number of resources. Other strengths of this study include using stakeholder feedback to choose the resources to show on the map. Finally, parents of positive outliers participated in designing the map. We believe that learning from these families who have reached a healthier weight despite living in obesogenic neighborhoods can inform effective interventions.

Our study had limitations. We interviewed only 11 parents and 10 community partners, so our resources may not be generalizable to other populations. We also used a large commercial database that often has misclassification errors, which required research staff hours for validation and maintenance. Although we validated many of the supermarkets and fitness centers, we were not able to validate the entire map. We also conducted our validation via telephone survey, which may not be as reliable as direct visualization of the business. In the future it may be helpful to have community members participate in this validation and to test the map or test the community resource map with a larger population. Finally, our results may not be generalizable to populations without access to health care or those located outside of Massachusetts.

Novel mapping tools can be used to tailor childhood obesity interventions and link families to healthy and affordable resources in their communities. The ability to validate resources is crucial to provide patients and their families with accurate information on community resources, and engaging key community partners in development can enhance their usability and salience.
